# Major and trace-element geochemistry of Late Cretaceous clastic rocks in the Jitai Basin, southeast China

**DOI:** 10.1038/s41598-021-93125-8

**Published:** 2021-07-05

**Authors:** Kai Yan, Chun-lian Wang, Steffen Mischke, Jiu-yi Wang, Li-jian Shen, Xiao-can Yu, Ling-yang Meng

**Affiliations:** 1 MNR Key Laboratory of Metallogeny and Mineral Assessment, Institute of Mineral Resources, Chinese Academy of Geological Sciences, Beijing, 100037 China; 2 Institute of Earth Sciences, University of Iceland, Sturlugata 7, 102 Reykjavík, Iceland; 3 902 Geological Brigade of Jiangxi Bureau of Exploration and Development for Geology and Mineral Resources, Xinyu, 338099 Jiangxi China

**Keywords:** Palaeoclimate, Geochemistry

## Abstract

Major, trace and rare earth element (REE) geochemistry of the late Cretaceous lower Zhoutian Formation from the Jitai Basin of Southeast China were measured by inductively coupled plasma mass spectrometry (ICP-MS) analysis to infer the provenance of the sediments and to reconstruct the palaeoenvironment and palaeoclimate. The wide range of Sr/Cu ratios point to a fluctuating palaeoclimate, and the negative correlation between the FeO/MnO and Al_2_O_3_/MgO ratios and the Sr/Cu ratio indicates that the late Cretaceous climate during the lower Zhoutian Formation in the Jitai Basin can be divided into two parts. The lower part experienced two cooling periods, whilst the upper part was dominated by warm-humid climate. Mostly corresponding trends of the B/Ga, Sr/Ba and Sr/Cu ratios show that the salinity changed consistently with the late Cretaceous climate during the lower Zhoutian Formation in the Jitai Basin. During the lower part, the salinity changed from salt water to fresh/brackish water. In the upper part, water was mainly fresh/brackish, and there were many changes from fresh/brackish water to salt water. The relatively stable Ni/Co, V/Cr, V/(V + Ni) and Ce/Ce* data indicate a long period of oxic conditions. The La-Th-Sc, Th-Sc-Zr/10 and La/Th-Hf data of the silt- and sandstones of the lower Zhoutian Formation show that its provenance was mainly a mixture of felsic upper crust sediments and older sedimentary rocks.

## Introduction

As an important carrier of geological information, the geochemical characteristics of clastic rocks record the significant information of provenance, structure, environment and ecological evolution in a reliable and detailed way. During the process of deposition, the distribution, circulation and differentiation (deficit and enrichment) of trace elements sensitive to redox conditions in water and sediments are not only related to their own chemical properties, but also controlled by the physical and chemical conditions of the deposition medium and the palaeoclimate conditions^1–4^. Hence, some major and trace elements that dissolve in water are sensitive to climatic change, and they can be used as a valuable proxies of palaeoclimate evolution^5^.

The Mesozoic was a period of drastic tectonic changes in South China and even the whole East Asian continent, and it was also a crucial period in the development of East Asian tectonics^6–10^. During the late Cretaceous-Paleogene, a series of rift basins were formed in the central part of South China, such as the Jianghan and Jitai basins. At this time, most of the lakes in these basins evolved into saline lakes and deposited huge quantities of halite and other saline minerals^11–12^. Deep brine is found in the late Cretaceous strata of the Zhoutian Formation in the Jitai Basin, which is rich in potassium, lithium, boron, rubidium, cesium, bromine, iodine and other high-value and emerging strategic mineral resources^13,14^.

The formation of deep brine deposits is controlled by the material sources, and the tectonic and climatic conditions during the sediment-formation period^15–17^. So far, basic geological research is rarely conducted in the Jitai Basin, and the existing data are not sufficient to study the formation mechanisms of the deep brine. In order to accumulate more geological information and to better understand the late Cretaceous characteristics of the Jitai Basin, we conducted geochemical analyses of major, trace and rare elements of silty mudstones and calcilutites of the lower Zhoutian Formation. Based on our geochemical data and previous studies, we discuss the palaeoclimate and the palaeoenvironmental characteristics and provenance of the late Cretaceous lower Zhoutian Formation in the Jitai Basin.

## Materials and methods

### Study area

**Figure 1:**
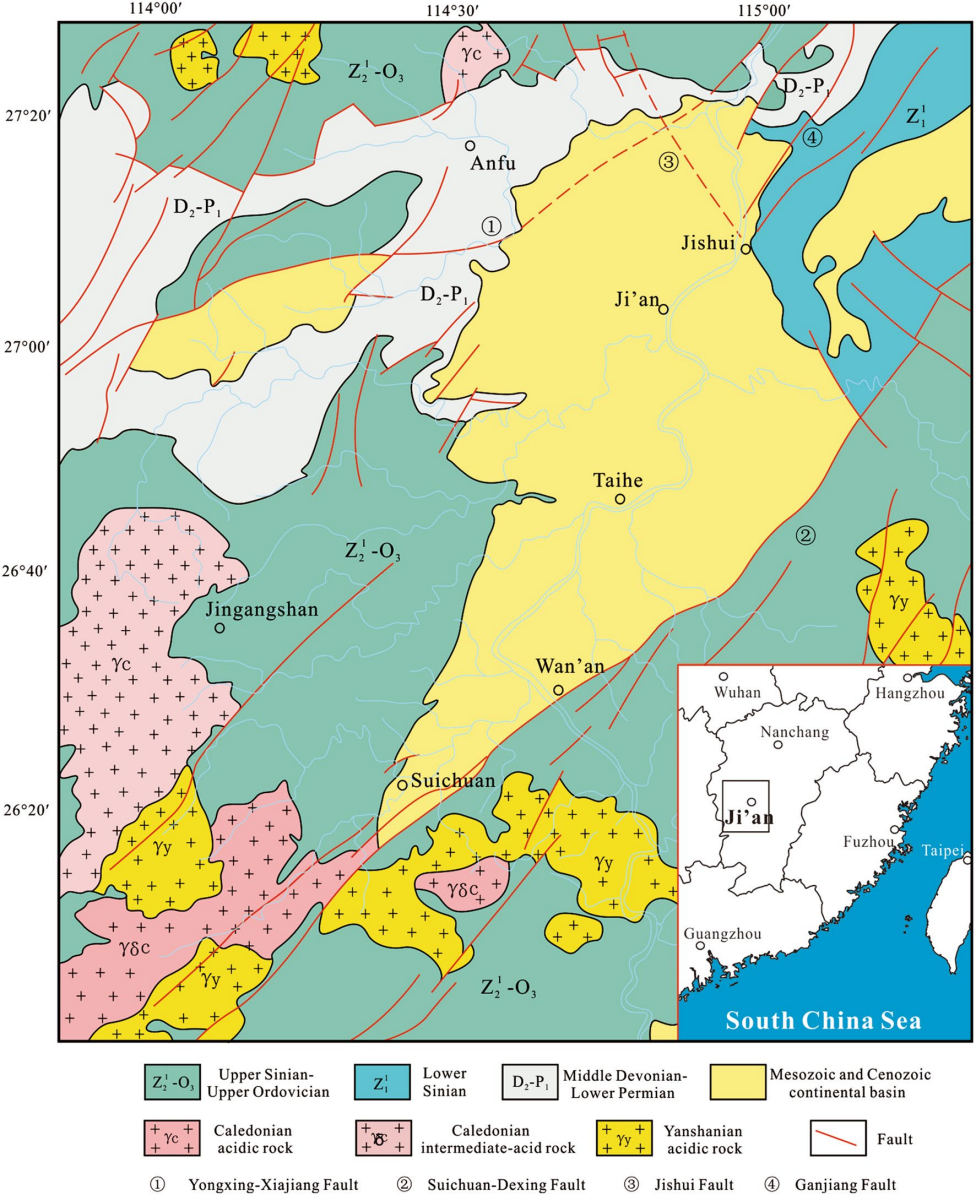
Generalized map of the Jitai Basin, Southeast China.

**Figure 2:**
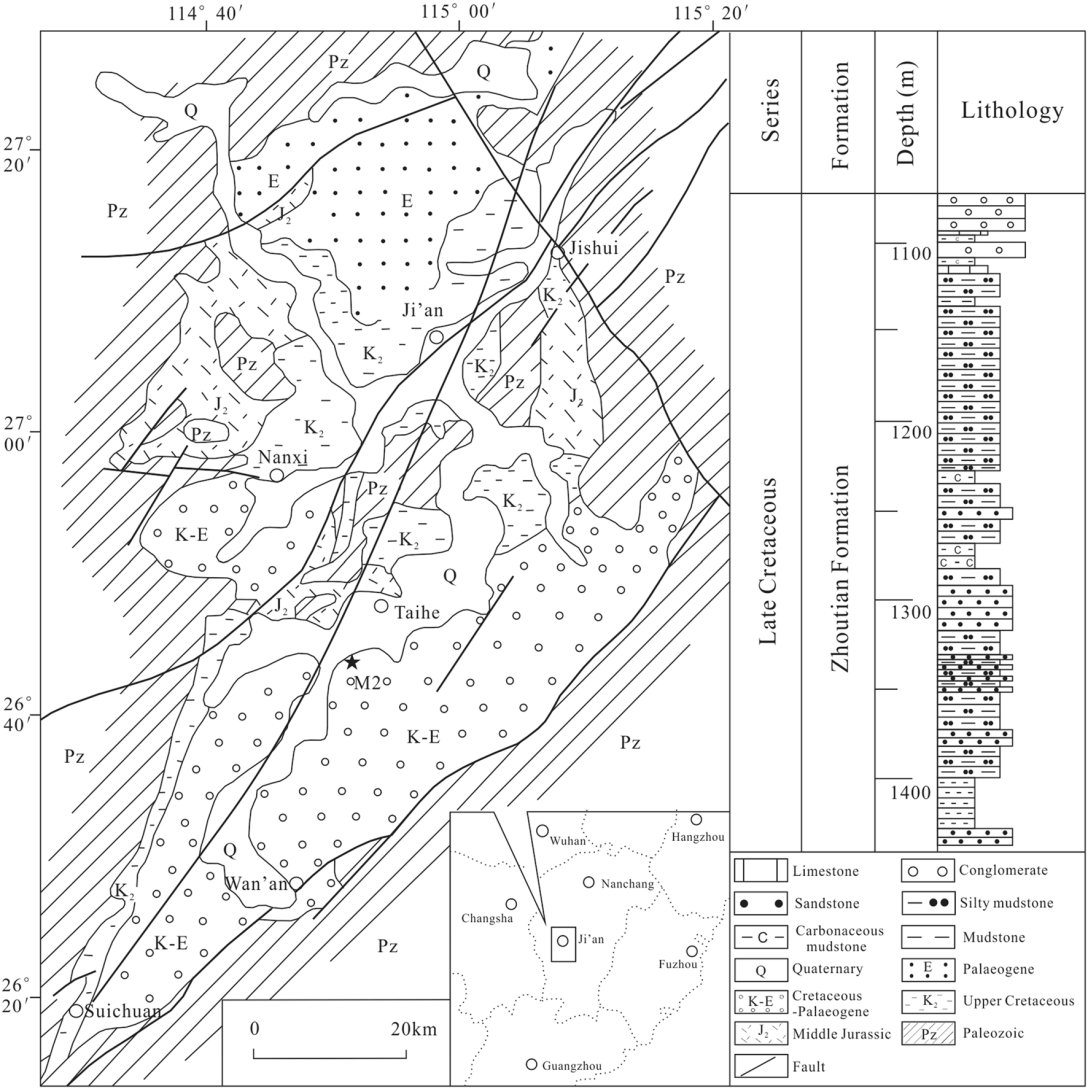
Simplified geological map of the Jitai Basin, Southeast China (modified from Yu et al.^18^).

The Jitai Basin is located in the transition zone between central uplift and southwest depression of Jiangxi Province. The basin is about 120 km long and 10–30 km wide, with an area of about 1850 km^2^^18^. The basement of the basin varies from region to region with late Paleozoic strata dominating in its northern part and early Paleozoic epimetamorphic series in its southern part (Fig. 1). There is a set of Cretaceous continental red strata in the basin with a thickness of several thousand meters. The lower Cretaceous is exposed at the western edge of the basin. The lower strata are mainly composed of magenta-coloured coarse clastic formations, the middle strata are mainly composed of magenta-coloured medium fine clastic formations, and the upper strata mainly include argillaceous deposits, forming a sedimentary cycle from coarse to fine. The upper Cretaceous is composed of magenta-coloured coarse clastic formations and medium fine clastic formations^19^. Lithium-rich ore occurs in the Zhoutian Formation of the late Cretaceous^20^. Basin development in the Jitai region was initiated in the late Cretaceous due to the intensification of the Yanshan movement and the strong influence of the Suichuan-Wan’an fault (Fig. 2).

### Sampling and methods

**Figure 3:**
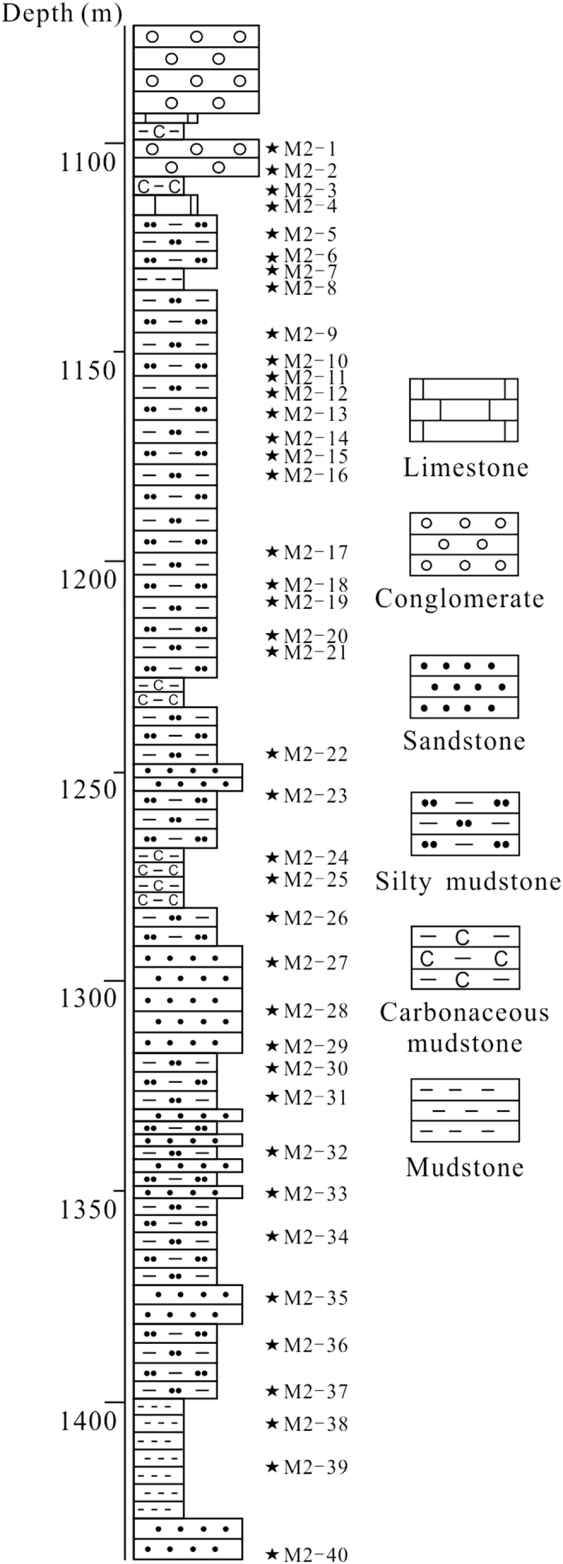
Stratigraphic column of the lower Zhoutian Formation in M2.

The samples were collected for this research from Well M2 in south Jitai Basin (Fig. 2). A total of 40 core samples from depths between 1100 to 1435 m were collected from the lower Zhoutian Formation (Fig. 3).

X-ray diffraction (XRD) was used to investigate the mineralogical characteristics of the samples. For XRD and geochemical analyses, samples were crushed and ground to less than 200 mesh (74 µm). XRD patterns were obtained using a Rigaku D/max-rA with Cu-kα radiation and Ni-filter in the microstructure analytical laboratory of Peking University. Whole-rock randomly oriented powders were scanned from 3°–70° 2θ, with a 0.02° 2θ step size.

X-ray fluorescence spectrometry was used to determine oxides of major elements such as SiO_2_, TiO_2_, Al_2_O_3_, Fe_2_O_3_, FeO, MnO, MgO, CaO, Na_2_O, K_2_O and P_2_O_5_. The powdered samples were first heated and then fused into glass sheets with a mixture of anhydrous lithium tetraborate, lithium fluoride and ammonium nitrate, and heated in a muffle furnace to determine loss on ignition. The fused samples were heated to 800 °C and analyzed using a sequential X-ray fluorescence spectrometer (AB104L, Axios-mAX). Calibrations of accuracy and reproducibility were conducted using the GB/T 14506.14-2010 and GB/T 14506.28-2010 standards.

An inductively coupled plasma mass spectrometer (ICP-MS, ELEMENT XR) was used to determine the contents of trace and REE. Powdered samples were heated with HF and HNO_3_. After evaporation to dryness, HNO_3_ was added to dissolve the samples. HNO_3_ was added to the beaker again before heating to 130 °C. Finally, the solution was diluted by distilled water for trace-element analyses. All geochemical analyses were carried out in the Analytical Laboratory Beijing Research Institute of Uranium Geology.

## Results

### Mineralogical composition

The whole-rock mineralogical compositions of the analyzed samples vary greatly (Table 1). Coal was not found in the 1435–1284-m segment, whilst most samples from the 1284–1100-m segment contain coal (4–21%) and pyrite (1–10%). The carbonate content (especially dolomite: 10–30%) is high in most samples beneath 1270 m core depth (Table 1). In contrast, carbonate is absent from most samples above. According to the carbonate content, the samples can be divided into carbonate and non-carbonate.

**Table 1:** XRD results of the Late Cretaceous samples from M2, Jitai Basin (unit in %).

No	Quartz	Feldspar	Dolomite	Calcite	Muscovite	Coal	Pyrite	Total clay
M2-1	51	22	0	0	22	0	0	5
M2-3	38	24	0	0	5	21	5	7
M2-5	30	10	15	22	8	10	2	3
M2-7	45	15	0	0	21	9	6	4
M2-9	54	15	0	0	23	5	0	3
M2-11	51	15	0	0	24	5	2	3
M2-13	49	16	0	0	20	7	4	4
M2-15	48	13	0	0	26	7	3	3
M2-17	51	14	0	0	21	7	3	4
M2-19	35	8	11	15	7	9	10	5
M2-21	49	10	3	0	16	15	3	4
M2-23	44	13	7	0	17	9	6	4
M2-25	15	6	20	15	5	17	7	15
M2-27	60	14	0	0	24	0	0	2
M2-29	58	23	0	5	12	0	0	2
M2-31	30	15	20	10	20	0	0	5
M2-33	65	10	5	0	17	0	0	3
M2-35	53	10	10	5	20	0	0	2
M2-37	50	9	15	10	12	0	0	4
M2-39	20	5	30	20	5	0	5	15

### Major elements

**Figure 4:**
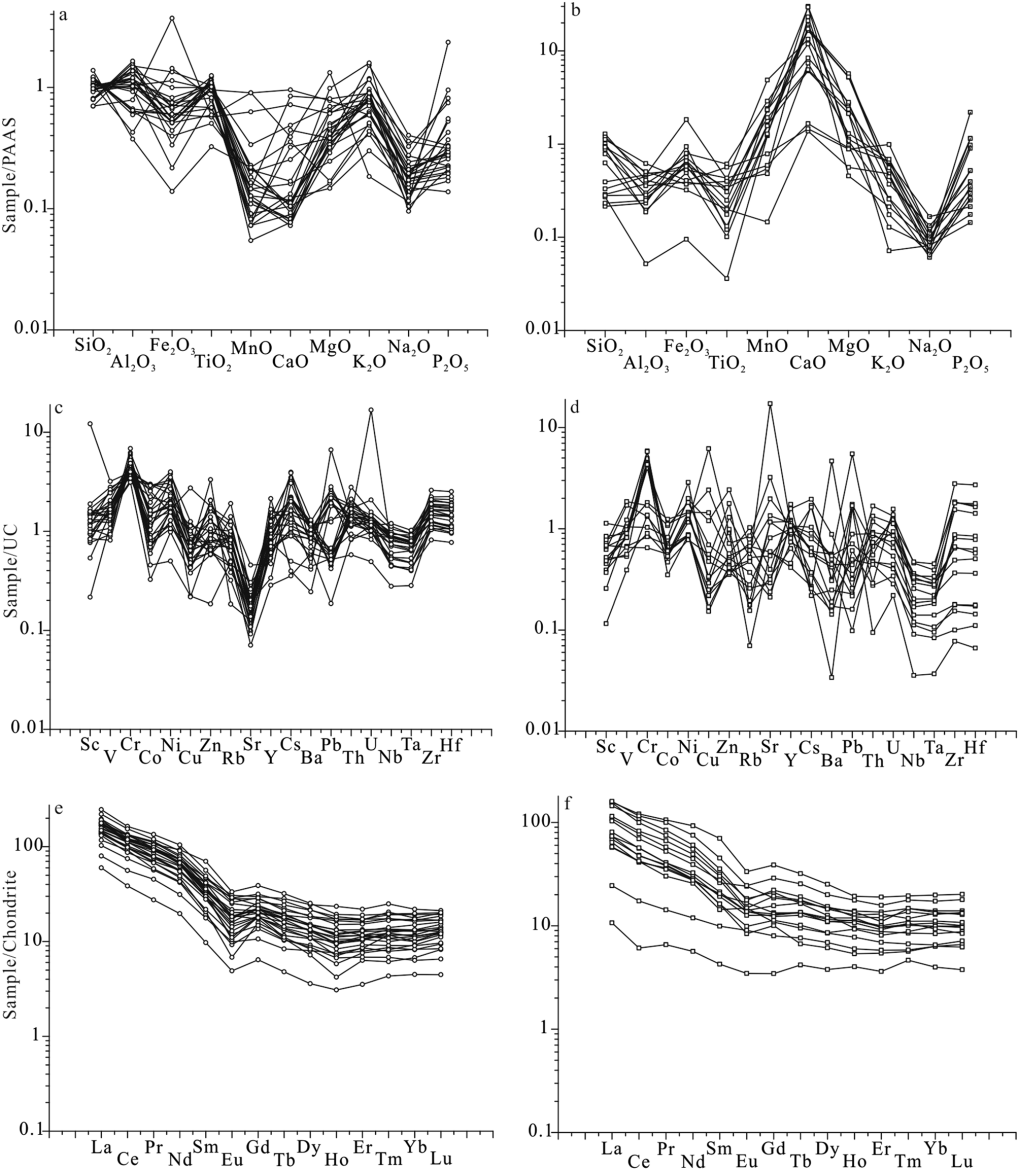
(**a**) PAAS-normalized major element diagram of non-carbonate samples in Well M2; (**b**) PAAS-normalized major-element diagram of carbonate samples in Well M2; (**c**) UC-normalized trace-element diagram of non-carbonate samples in Well M2; (**d**) UC-normalized trace-element diagram of carbonate samples in Well M2; (**e**) Chondrite-normalized rare element diagram of non-carbonate samples in Well M2; (**f**) Chondrite-normalized rare element diagram of carbonate samples in Well M2 (modified after Evensen et al.^22^) (original data are given in Tables 2, 3 and 4).

The SiO_2_ contents are the highest in most sample, and they vary over a large range from 13.48 to 86.48% (Table 2). The Al_2_O_3_ contents are between 0.981 and 31.12%, and the Fe_2_O_3_ contents between 0.685 and 26.81%. The contents of CaO and MnO from non-carbonate samples in Well M2 are significantly lower in comparison to the Average Post-Archean Australian Shale (PAAS)^21^, whilst CaO contents of carbonate samples in Well M2 are higher. The Na_2_O contents of all samples are lower than those of PAAS and show strong depletion in the plot with values normalized to PAAS (Fig. 4a,b). The Sr/Cu ratios range from 0.99 to 106.56, with an average of 12.89 (Table 5). The FeO/MnO ratios range from 4.15 to 412.22, with an average of 125.51. The Al_2_O_3_/MgO ratios vary between 0.21 and 63.48, with an average of 15.73. The Mg/Ca ratios range from 0.04 to 13.95, with an average of 3.89.

**Table 2:** Contents of major elements from Well M2, Jitai Basin (unit in %).

No	SiO_2_	Al_2_O_3_	Fe_2_O_3_	TiO_2_	MnO	CaO	MgO	K_2_O	Na_2_O	P_2_O_5_	FeO	LOI	Total
M2-1	66.74	18.34	1.56	0.786	0.008	0.58	0.83	4.36	0.327	0.036	0.65	6.34	100.56
M2-2	20.75	8.51	4.27	0.341	0.060	30.13	2.48	2.37	0.200	0.034	1.06	30.30	100.51
M2-3	76.03	11.95	4.34	0.672	0.016	0.21	0.54	1.51	0.165	0.027	2.60	4.54	102.60
M2-4	17.00	0.981	0.69	0.036	0.135	39.04	4.69	0.26	0.098	0.023	0.56	36.69	100.20
M2-5	39.39	3.70	3.56	0.101	0.138	27.28	1.00	0.78	0.103	0.040	1.99	23.41	101.50
M2-6	75.32	11.22	5.13	0.680	0.014	0.15	0.72	1.80	0.179	0.022	3.60	4.35	103.19
M2-7	43.87	28.36	9.66	1.040	0.023	0.22	1.41	4.28	0.390	0.119	6.35	10.53	106.25
M2-8	44.00	14.90	26.81	0.580	0.099	0.51	2.91	0.68	0.137	0.376	19.24	8.43	118.67
M2-9	62.57	22.31	3.68	1.070	0.013	0.09	0.81	3.04	0.267	0.043	2.72	6.06	102.67
M2-10	66.59	19.79	3.76	1.000	0.018	0.15	0.97	2.96	0.249	0.046	2.71	4.41	102.65
M2-11	60.71	22.10	5.45	1.040	0.020	0.14	1.36	2.79	0.229	0.036	3.87	6.07	103.81
M2-12	69.22	17.42	3.76	0.952	0.012	0.11	0.77	2.32	0.210	0.032	2.50	5.15	102.45
M2-13	59.93	21.17	5.93	1.050	0.024	0.13	1.10	2.79	0.229	0.041	3.67	7.41	103.48
M2-14	58.36	25.79	3.94	1.150	0.013	0.11	0.74	2.99	0.232	0.051	2.72	6.57	102.67
M2-15	50.89	28.00	3.95	1.100	0.008	0.12	0.70	3.57	0.292	0.044	1.37	11.30	101.34
M2-16	64.90	21.18	3.67	0.997	0.016	0.15	0.84	2.24	0.201	0.031	2.33	5.76	102.31
M2-17	58.55	24.53	4.95	1.090	0.014	0.10	1.01	2.60	0.252	0.047	3.31	6.86	103.31
M2-18	49.10	5.62	5.75	0.610	0.318	15.38	2.38	0.97	0.111	0.028	4.88	19.26	104.40
M2-19	24.59	8.85	13.21	0.419	0.535	17.37	5.90	1.37	0.160	0.063	11.31	27.04	110.82
M2-20	65.65	8.05	10.36	0.823	0.008	0.16	0.32	1.11	0.191	0.029	1.76	13.20	101.66
M2-21	65.17	19.01	4.77	0.934	0.025	0.12	0.91	2.18	0.211	0.035	3.46	6.56	103.39
M2-22	49.73	29.83	4.91	1.220	0.006	0.10	0.63	3.36	0.432	0.050	2.08	9.72	102.07
M2-23	57.59	19.52	8.20	0.954	0.099	1.24	1.71	2.18	0.206	0.152	5.14	8.12	105.11
M2-24	49.86	25.77	7.19	0.998	0.037	0.63	1.53	2.95	0.330	0.130	4.75	10.53	104.71
M2-25	13.48	4.41	6.78	0.121	0.264	38.24	2.11	0.47	0.095	0.350	5.04	33.19	104.55
M2-26	64.78	22.60	3.17	1.010	0.010	0.14	0.36	1.61	0.234	0.068	2.08	6.00	102.05
M2-27	50.20	31.12	2.41	1.250	0.009	0.42	0.84	5.64	0.483	0.053	1.34	7.49	101.25
M2-28	86.46	7.08	1.00	0.324	0.024	0.46	0.37	1.88	0.125	0.034	0.73	2.14	100.63
M2-29	73.71	12.58	4.33	0.568	0.069	0.93	1.34	3.39	0.147	0.084	2.46	2.37	101.98
M2-30	76.59	12.04	2.86	0.504	0.022	0.33	1.06	3.07	0.139	0.088	2.22	2.88	101.80
M2-31	18.10	7.06	5.18	0.307	0.253	21.50	11.45	2.11	0.125	0.052	4.18	33.33	103.65
M2-32	55.15	3.53	4.78	0.249	0.257	10.92	6.16	0.65	0.079	0.047	3.89	18.13	103.84
M2-33	80.50	7.75	2.32	0.199	0.016	1.79	1.24	1.77	0.085	0.144	1.87	3.76	101.44
M2-34	74.54	9.13	3.81	0.432	0.065	2.15	2.00	2.41	0.107	0.156	2.91	5.10	102.81
M2-35	61.24	19.26	5.40	0.818	0.009	0.17	2.16	5.88	0.154	0.059	3.71	4.78	103.64
M2-36	59.29	6.75	2.82	0.342	0.211	8.17	5.17	2.06	0.098	0.184	2.47	14.86	102.43
M2-37	72.60	12.19	3.69	0.592	0.016	1.10	1.77	3.31	0.114	0.052	2.83	4.10	102.36
M2-38	58.21	9.54	2.67	0.397	0.053	9.59	2.85	2.54	0.138	0.083	1.27	13.36	100.70
M2-39	14.57	4.70	4.77	0.131	0.141	22.26	11.52	1.65	0.078	0.056	3.63	40.03	103.54
M2-40	70.15	11.71	2.93	0.565	0.086	1.90	1.94	3.66	0.133	0.183	2.34	6.69	102.29

### Trace elements

The three trace elements with the highest average content are Ba, Zr and Sr, and their average contents are 451.20 ppm, 255.93 ppm and 148.85 ppm, respectively (Table 3). The contents of various trace elements vary over a large range if they are normalized to Upper Crust (UC) data (Fig. 4c,d)^21^. The Cr contents are enriched relative to UC in most non-carbonate samples, but Sr contents show strong depletion (Fig. 4c). In contrast, Cr contents of some carbonate samples are enriched (Fig. 4d). The B/Ga ratios range from 1.03 to 42.78, with an average of 6.80 (Table 5). The Sr/Ba ratios range from 0.04 to 11.13, with an average of 0.99. The Ni/Co ratios range from 1.23 to 6.97, with an average of 3.48. The V/Cr ratios range from 0.24 to 2.21, with an average of 0.75, and the V/(V + Ni) ratios from 0.43 to 0.88, with an average of 0.70.

**Table 3:** Contents of trace elements from Well M2, Jitai Basin (unit in ppm).

No	Sc	V	Cr	Co	Ni	Cu	Zn	Ga	Rb	Sr	Y	Cs	Ba	Pb	Th	U	Nb	Ta	Zr	Hf	B
M2-1	12.9	85.8	143	5.95	32.7	68.3	126	24.0	140.0	67.4	7.4	11.3	540	34.4	15.0	47.00	17.6	1.39	313	8.31	79.5
M2-2	6.8	102.0	46	7.13	38.7	29.9	172	11.5	75.0	205.0	10.0	6.3	173	26.1	7.3	3.45	7.9	0.64	93	2.92	34.8
M2-3	9.2	48.6	168	9.08	23.2	13.3	43	14.6	48.4	45.3	13.9	3.6	450	9.4	10.0	2.31	13.6	1.06	199	6.19	52.0
M2-4	1.3	23.4	48	5.28	23.2	155.0	65	1.7	7.8	207.0	9.1	1.0	19	24.1	1.0	0.61	0.9	0.08	15	0.39	3.1
M2-5	4.3	40.1	29	6.89	39.7	12.7	126	5.5	19.7	117.0	14.0	1.3	79	9.1	2.9	0.95	2.3	0.18	19	0.64	10.1
M2-6	11.7	53.5	206	7.93	23.3	12.6	146	14.9	56.3	34.5	14.8	3.8	290	32.0	8.8	2.75	13.6	1.17	307	9.28	89.8
M2-7	15.9	149.0	133	29.90	53.5	24.6	113	35.5	129.0	72.4	30.5	12.9	563	34.4	19.7	4.03	22.3	1.67	233	6.34	60.7
M2-8	20.7	167.0	122	29.00	66.0	16.3	236	23.6	20.6	44.1	46.9	1.5	134	26.9	15.8	2.80	11.0	0.93	155	4.46	24.4
M2-9	17.0	86.2	188	15.10	36.3	15.7	57	26.2	96.0	56.6	28.4	7.6	570	9.8	17.7	3.73	21.7	1.66	343	10.10	78.7
M2-10	16.2	85.0	154	8.60	32.3	11.1	69	26.3	97.6	60.1	31.6	5.4	669	6.7	15.1	3.49	19.9	1.62	403	12.10	99.9
M2-11	17.5	103.0	163	14.60	39.3	15.6	91	31.6	95.2	49.3	18.3	8.2	638	8.6	13.2	2.83	21.6	1.69	273	8.23	74.0
M2-12	14.3	78.6	194	13.30	31.5	16.0	56	21.3	73.9	48.4	25.6	4.2	482	10.1	13.3	3.18	18.3	1.50	290	8.12	75.4
M2-13	16.3	96.8	113	22.30	38.2	28.2	71	27.9	88.1	57.0	17.4	6.2	610	19.8	16.0	3.63	20.8	1.61	381	11.30	97.9
M2-14	13.3	114.0	145	6.87	25.7	12.8	58	30.5	90.7	58.1	20.2	7.9	656	6.8	19.0	3.79	24.2	1.90	317	9.42	90.2
M2-15	12.5	158.0	138	29.50	47.4	23.7	50	34.7	78.1	40.7	19.2	8.0	691	39.5	12.0	3.75	24.4	1.84	275	8.12	80.0
M2-16	10.3	109.0	162	15.00	35.1	10.7	65	26.1	70.8	38.0	13.3	3.9	489	8.4	14.7	2.53	21.6	1.76	234	6.64	92.4
M2-17	16.8	110.0	156	16.50	62.3	20.5	89	34.8	94.2	64.4	34.2	5.9	707	9.0	29.8	4.36	26.4	2.06	421	13.00	116.0
M2-18	7.3	49.5	140	5.90	16.9	5.4	26	7.5	28.8	102.0	38.3	0.9	2562	4.8	17.9	3.72	11.4	0.86	529	15.70	93.5
M2-19	12.4	62.3	63	12.40	28.9	8.2	36	11.3	41.3	73.6	26.7	2.2	308	5.3	7.1	2.61	9.0	0.67	122	3.62	32.2
M2-20	8.4	55.8	217	19.50	75.3	31.2	48	14.2	35.7	32.2	17.0	1.8	227	99.8	15.1	3.62	15.0	1.39	492	14.60	178.0
M2-21	14.0	73.0	144	11.90	34.1	14.2	145	25.3	71.3	49.9	36.7	4.6	543	7.1	16.5	3.84	20.6	1.62	397	11.10	101.0
M2-22	8.8	106.0	129	26.00	79.4	19.1	57	34.0	85.8	70.4	22.1	7.8	568	32.5	18.2	5.80	28.0	2.06	286	8.43	89.3
M2-23	134.0	191.0	138	28.90	61.4	19.5	99	27.2	42.8	64.6	16.8	4.9	517	29.3	14.2	3.35	22.0	1.71	337	9.74	86.0
M2-24	18.6	146.0	120	11.30	43.8	17.4	121	33.2	107.0	101.0	27.6	7.8	645	18.6	22.4	3.62	22.5	1.75	264	7.42	79.7
M2-25	5.0	62.5	36	8.23	57.4	7.2	70	7.3	17.4	407.0	18.3	0.9	85	25.3	3.1	4.36	2.8	0.21	34	1.00	19.2
M2-26	5.9	91.5	167	6.95	31.9	12.0	44	26.0	52.4	106.0	11.7	6.0	242	8.0	14.1	4.03	23.2	2.01	350	10.50	104.0
M2-27	10.3	93.4	109	3.25	19.6	9.4	41	41.5	157.0	159.0	10.3	14.6	383	8.5	22.5	3.19	29.9	2.24	357	10.60	133.0
M2-28	2.4	70.4	240	4.55	10.0	5.5	13	5.8	55.0	24.8	6.3	1.3	329	7.8	6.2	1.38	6.9	0.62	217	6.02	84.4
M2-29	9.1	99.7	161	25.30	31.1	25.1	42	14.5	105.0	48.2	20.7	3.3	465	28.8	12.1	3.46	11.4	0.88	222	6.07	128.0
M2-30	15.3	123.0	182	7.48	21.2	19.0	46	13.7	91.1	61.5	15.4	3.3	344	2.8	8.9	3.20	11.1	0.89	196	5.51	102.0
M2-31	8.0	111.0	59	10.70	36.2	17.3	34	9.5	80.8	691.0	23.6	3.3	298	82.3	5.9	3.84	6.4	0.48	69	2.10	66.4
M2-32	2.8	57.6	166	6.60	17.4	3.8	31	3.9	21.7	406.0	26.0	0.8	136	3.4	9.1	2.04	5.1	0.44	347	9.84	48.7
M2-33	4.1	73.1	205	4.66	15.5	13.9	29	8.9	54.5	82.6	16.2	2.0	229	1.5	7.8	1.50	4.6	0.43	165	4.89	68.4
M2-34	5.7	49.2	203	5.63	29.1	36.0	38	9.6	75.1	110.0	23.5	3.8	273	3.2	12.5	2.43	8.5	0.74	349	9.55	123.0
M2-35	17.4	85.4	139	10.30	70.1	5.4	59	26.9	213.0	85.9	22.4	14.3	712	6.3	17.9	2.90	18.4	1.43	206	5.63	204.0
M2-36	5.3	48.9	150	3.49	17.3	6.2	25	7.6	68.1	470.0	24.9	2.2	244	6.6	9.8	2.38	7.3	0.59	292	8.22	106.0
M2-37	10.8	64.4	170	16.20	45.4	17.6	75	15.0	112.0	90.9	19.2	7.4	425	42.1	12.4	2.83	13.1	1.05	241	6.44	119.0
M2-38	8.6	58.6	139	11.10	28.1	60.3	51	9.7	95.1	1126.0	17.7	6.0	168	13.2	9.5	1.81	8.2	0.61	126	3.32	415.0
M2-39	5.3	33.4	31	5.34	27.1	8.4	92	6.0	58.2	188.0	21.0	2.4	104	7.4	4.7	0.79	3.0	0.24	29	0.83	87.5
M2-40	9.2	53.8	151	6.01	17.1	5.5	40	15.0	115.0	138.0	34.6	7.2	482	3.9	14.2	3.14	11.7	1.00	340	10.30	204.0

### Rare elements

The three most abundant REE are Ce, La and Nd, with the Ce content ranging from 4.88 to 131 ppm, the La content ranging from 3.2 to 73.9 ppm, and the Nd content ranging from 3.4 to 62.7 ppm (Table 4). If normalized to chondrite values, all samples of the Zhoutian Formation show a set of steep dips of the light REE (LREE) curves, whilst the curves of the heavy REE (HREE) are flat (Fig. 4e,f)^22^. For the non-carbonate samples, Eu contents represent negative anomalies, and Eu curves are V-shaped in the standardized diagram (Fig. 4e). For the samples of carbonate, Eu contents show slightly negative anomalies (Fig. 4f). The ƩREE values range from 17.80 to 323.31, with an average of 181.14 (Table 4). The Ce/Ce* ratios range from 0.70 to 1.07, with an average of 0.91 (Table 5). The Th/Sc ratios range from 0.58 to 3.23, with an average of 1.31, and the La/Sc ratios from 0.33 to 11.51, with an average of 4.20.

**Table 4:** Contents of rare elements from Well M2, Jitai Basin (unit in ppm).

No	La	Ce	Pr	Nd	Sm	Eu	Gd	Tb	Dy	Ho	Er	Tm	Yb	Lu	ƩREE
M2-1	49.9	89.8	10.30	38.2	6.77	1.30	4.5	0.54	2.3	0.30	1.3	0.18	1.42	0.247	207.09
M2-2	20.7	38.4	4.60	17.0	3.22	0.59	2.6	0.33	2.0	0.38	1.1	0.17	1.33	0.198	92.60
M2-3	30.9	59.9	6.80	25.1	3.84	0.65	3.5	0.51	2.9	0.52	1.7	0.26	1.72	0.283	138.62
M2-4	3.2	4.9	0.79	3.4	0.85	0.24	0.9	0.21	1.2	0.28	0.8	0.14	0.84	0.113	17.80
M2-5	7.3	13.9	1.71	7.2	1.98	0.63	2.1	0.38	2.2	0.42	1.2	0.18	1.33	0.187	40.70
M2-6	23.9	44.9	5.43	18.8	3.54	0.69	2.8	0.42	2.6	0.53	1.8	0.35	2.11	0.374	108.15
M2-7	58.6	108.0	13.60	49.1	9.30	1.98	7.4	1.12	5.9	1.21	3.6	0.58	4.04	0.581	264.95
M2-8	46.3	96.6	11.50	43.6	9.40	2.15	7.7	1.42	7.9	1.64	4.6	0.75	4.60	0.635	238.76
M2-9	53.4	96.6	11.30	39.4	6.46	1.12	5.8	0.95	5.7	1.16	3.4	0.52	3.52	0.557	229.79
M2-10	47.1	92.4	10.60	40.1	7.03	1.58	6.2	0.96	5.2	1.05	3.2	0.53	3.28	0.504	219.78
M2-11	50.4	106.0	11.70	44.2	7.47	1.26	5.7	0.79	4.2	0.67	2.4	0.32	2.09	0.353	237.60
M2-12	40.8	78.2	8.40	33.9	5.86	1.26	5.2	0.88	4.8	0.93	2.4	0.40	2.75	0.423	186.16
M2-13	49.8	92.7	11.30	39.7	6.86	1.52	5.5	0.69	3.8	0.65	2.3	0.35	2.47	0.418	218.00
M2-14	54.5	106.0	13.30	47.1	8.36	1.30	5.8	0.89	4.7	0.83	2.7	0.39	2.69	0.401	248.95
M2-15	41.2	81.6	9.49	37.3	8.27	1.76	6.0	0.98	4.8	0.81	2.4	0.36	2.39	0.372	197.73
M2-16	42.6	77.4	9.45	34.0	6.29	0.73	4.7	0.65	3.5	0.50	1.8	0.24	1.74	0.252	183.86
M2-17	73.9	131.0	16.20	62.7	11.30	2.02	8.2	1.30	7.0	1.20	3.6	0.61	3.72	0.599	323.31
M2-18	47.1	96.7	12.70	55.7	14.00	2.34	10.1	1.60	8.1	1.35	4.0	0.58	4.16	0.605	259.00
M2-19	32.6	62.2	6.95	26.8	5.22	1.69	4.8	0.87	4.7	0.97	2.9	0.44	2.93	0.419	153.47
M2-20	41.3	77.9	8.18	28.2	4.13	0.48	4.1	0.58	3.4	0.71	2.2	0.37	2.79	0.456	174.78
M2-21	52.3	92.0	11.70	41.2	7.43	1.20	6.8	1.21	6.8	1.28	3.6	0.52	3.43	0.506	230.03
M2-22	52.7	105.0	12.50	44.5	8.29	2.03	6.3	0.95	4.9	0.93	2.8	0.41	2.79	0.381	244.41
M2-23	44.0	88.8	10.90	41.2	7.12	1.28	5.7	0.86	4.1	0.67	2.2	0.37	2.39	0.368	209.94
M2-24	66.0	123.0	14.40	54.3	9.82	1.82	7.5	1.06	6.0	1.06	3.2	0.50	3.61	0.503	292.79
M2-25	17.2	33.6	3.63	15.5	2.87	1.04	2.8	0.47	2.7	0.54	1.5	0.20	1.37	0.214	83.64
M2-26	35.1	73.4	7.01	25.7	4.36	0.84	3.8	0.52	2.8	0.47	1.4	0.20	1.33	0.196	157.20
M2-27	57.4	98.2	11.80	39.3	5.96	0.77	4.8	0.55	2.7	0.41	1.6	0.25	1.93	0.334	225.99
M2-28	18.0	30.9	3.30	11.8	1.95	0.34	1.7	0.24	1.2	0.22	0.7	0.13	0.94	0.134	71.52
M2-29	43.1	78.8	9.12	33.2	5.94	1.07	4.8	0.80	4.2	0.78	2.5	0.33	2.37	0.379	187.28
M2-30	44.6	89.9	9.46	32.9	5.98	1.00	4.7	0.68	3.5	0.59	1.9	0.27	1.90	0.289	197.67
M2-31	46.6	79.8	8.97	33.0	6.32	1.28	5.5	0.82	4.0	0.66	1.9	0.26	1.76	0.263	191.10
M2-32	19.3	33.0	4.35	16.8	3.96	1.17	3.5	0.67	3.7	0.78	2.1	0.31	2.23	0.301	92.09
M2-33	31.0	55.7	6.29	23.5	5.54	0.99	4.1	0.83	4.8	0.96	2.3	0.44	2.83	0.385	139.65
M2-34	43.4	87.4	10.10	36.2	7.10	1.24	5.8	0.95	4.9	0.85	2.5	0.41	2.71	0.405	203.95
M2-35	53.2	95.1	11.10	40.5	7.16	1.42	5.2	0.90	4.7	0.85	2.7	0.42	3.14	0.481	226.92
M2-36	34.4	65.9	7.99	29.5	6.03	1.04	5.0	0.89	4.7	0.89	2.7	0.35	2.77	0.396	162.57
M2-37	39.3	69.4	8.10	29.5	5.58	0.91	4.6	0.72	4.3	0.74	2.2	0.30	2.28	0.361	168.28
M2-38	22.1	37.9	4.68	17.1	3.25	0.69	2.9	0.51	2.8	0.65	1.7	0.26	2.08	0.290	96.90
M2-39	17.4	33.8	4.23	16.5	4.09	0.96	3.2	0.63	3.5	0.75	1.9	0.32	1.96	0.253	89.53
M2-40	48.0	92.4	12.00	45.2	9.05	1.71	7.5	1.27	6.4	1.24	3.3	0.53	3.64	0.538	232.83

**Table 5:** Selected element ratios from Well M2, Jitai Basin.

No	Sr/Cu	FeO/MnO	Al_2_O_3_/MgO	Mg/Ca	B/Ga	Sr/Ba	Ni/Co	V/Cr	V/(V + Ni)	Ce/Ce*	La/Sc	Th/Sc
M2-1	0.99	81.25	21.99	1.61	3.31	0.12	5.50	0.60	0.72	0.90	3.87	1.16
M2-2	6.86	17.67	3.43	0.09	3.03	1.18	5.43	2.21	0.72	0.90	3.03	1.06
M2-3	3.41	162.50	22.09	2.89	3.56	0.10	2.56	0.29	0.68	0.94	3.35	1.08
M2-4	1.34	4.15	0.21	0.13	1.90	11.13	4.39	0.49	0.50	0.70	2.52	0.80
M2-5	9.21	14.42	3.70	0.04	1.83	1.49	5.76	1.37	0.50	0.90	1.71	0.69
M2-6	2.74	257.14	15.58	5.23	6.03	0.12	2.94	0.26	0.70	0.90	2.04	0.75
M2-7	2.94	276.09	20.11	7.15	1.71	0.13	1.79	1.12	0.74	0.87	3.69	1.24
M2-8	2.71	194.34	5.12	6.36	1.03	0.33	2.28	1.37	0.72	0.96	2.24	0.76
M2-9	3.61	209.23	27.68	9.53	3.00	0.10	2.40	0.46	0.70	0.90	3.14	1.04
M2-10	5.41	150.56	20.49	7.25	3.80	0.10	3.76	0.55	0.72	0.95	2.91	0.93
M2-11	3.16	193.50	16.25	10.95	2.34	0.08	2.69	0.63	0.72	1.00	2.88	0.75
M2-12	3.03	208.33	22.65	8.06	3.54	0.10	2.37	0.41	0.71	0.96	2.85	0.93
M2-13	2.02	152.92	19.25	9.12	3.51	0.09	1.71	0.86	0.72	0.89	3.06	0.98
M2-14	4.54	209.23	34.76	7.71	2.96	0.09	3.74	0.79	0.82	0.90	4.10	1.43
M2-15	1.72	171.25	40.06	6.70	2.31	0.06	1.61	1.14	0.76	0.94	3.30	0.96
M2-16	3.55	145.63	25.33	6.41	3.54	0.08	2.34	0.67	0.75	0.88	4.14	1.43
M2-17	3.14	236.43	24.29	11.22	3.33	0.09	3.78	0.71	0.64	0.87	4.40	1.77
M2-18	18.82	15.35	2.36	0.17	12.50	0.04	2.86	0.35	0.75	0.90	6.46	2.46
M2-19	9.01	21.14	1.50	0.38	2.85	0.24	2.33	0.98	0.68	0.94	2.63	0.58
M2-20	1.03	220.00	24.92	2.26	12.54	0.14	3.86	0.26	0.43	0.97	4.91	1.79
M2-21	3.51	138.40	20.80	8.46	3.99	0.09	2.87	0.51	0.68	0.85	3.74	1.18
M2-22	3.69	346.67	47.05	7.04	2.63	0.12	3.05	0.82	0.43	0.94	6.01	2.08
M2-23	3.31	51.92	11.42	1.53	3.16	0.12	2.12	1.38	0.68	0.93	0.33	0.11
M2-24	5.80	128.38	16.84	2.69	2.40	0.16	3.88	1.22	0.57	0.91	3.55	1.20
M2-25	56.22	19.09	2.09	0.06	2.64	4.81	6.97	1.74	0.52	0.97	3.47	0.62
M2-26	8.83	208.00	63.48	2.91	4.00	0.44	4.59	0.55	0.76	1.07	5.96	2.39
M2-27	16.86	148.89	37.18	2.22	3.20	0.42	6.03	0.86	0.52	0.86	5.57	2.18
M2-28	4.49	30.42	19.08	0.89	14.63	0.08	2.19	0.29	0.74	0.91	7.59	2.60
M2-29	1.92	35.65	9.39	1.60	8.83	0.10	1.23	0.62	0.83	0.91	4.72	1.32
M2-30	3.24	100.91	11.36	3.57	7.45	0.18	2.83	0.68	0.88	1.00	2.92	0.58
M2-31	39.94	16.52	0.62	0.59	6.97	2.32	3.38	1.87	0.75	0.82	2.42	0.74
M2-32	106.56	15.14	0.57	0.63	12.58	2.99	2.64	0.35	0.77	0.91	10.99	3.23
M2-33	5.94	116.88	6.25	0.77	7.70	0.36	3.33	0.36	0.83	0.89	11.51	1.93
M2-34	3.06	44.77	4.57	1.03	12.80	0.40	5.17	0.24	0.63	0.95	7.59	2.19
M2-35	15.85	412.22	8.92	13.95	7.58	0.12	6.81	0.61	0.55	0.89	3.06	1.03
M2-36	75.68	11.71	1.31	0.70	14.00	1.93	4.96	0.33	0.74	0.91	6.53	1.86
M2-37	5.16	176.88	6.89	1.79	7.93	0.21	2.80	0.38	0.59	0.89	3.64	1.15
M2-38	18.67	23.96	3.35	0.33	42.78	6.70	2.53	0.42	0.68	0.85	2.58	1.11
M2-39	22.30	25.74	0.41	0.58	14.51	1.81	5.07	1.06	0.55	0.90	3.29	0.90
M2-40	25.27	27.21	6.04	1.13	13.60	0.29	2.85	0.36	0.76	0.88	5.19	1.54

## Discussion

### Palaeoclimate

The element geochemistry including the Sr/Cu, FeO/MnO, Al_2_O_3_/MgO and Mg/Ca ratios is an established tool for the reconstruction of past climate conditions^23–25^. The Sr/Cu ratio is a sensitive indicator of palaeoclimate, with high Sr/Cu ratios typically reflecting hot and arid climate, whilst low ratios indicate warm and humid climate^23^. According to Lerman^23^, Sr/Cu ratios between 1.3 and 5.0 suggest a warm-humid climate, while ratios greater than 5.0 indicate a hot-arid climate. Mn content is relatively high in dry environments, but low in relatively humid conditions where Fe is rapidly precipitated from colloidal iron hydroxides [Fe(OH)_3_]. Thus, high FeO/MnO ratios in sediments correspond to warm-humid climate and low ratios to hot-arid climate. The Al_2_O_3_/MgO ratios in clay minerals and their variation can also reflect the climate during deposition, with high ratios indicating warm-humid climate and low ratios indicating dry climate^24^. The Mg/Ca ratios are also very sensitive to climate change. In general, high Mg/Ca ratios indicate arid climate, whilst low ratios reflect humid conditions^25^.

**Figure 5:**
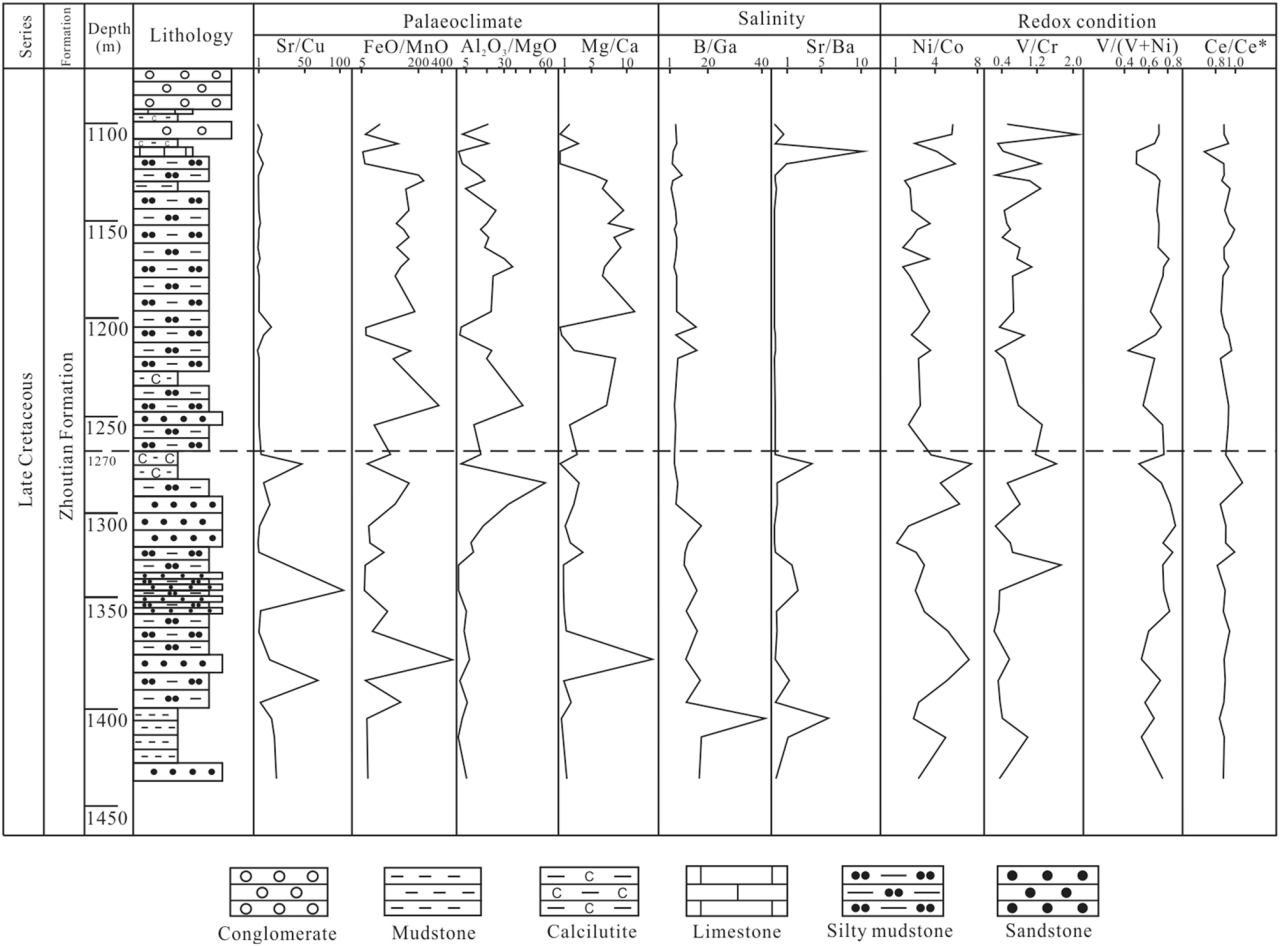
Element ratios from the lower Zhoutian Formation in the Jitai Basin as palaeoclimate, salinity and redox-condition proxies.

The wide range of Sr/Cu ratios between 0.99 to 106.56 shows that the Jitai Basin experienced relatively large climatic fluctuations during the late Cretaceous period (Table 5 and Fig. 5). Based on the variations of the Sr/Cu ratios, the late Cretaceous climate in the Jitai Basin is roughly divided into two parts, represented by the 1435–1270-m and 1270–1100-m core sections. The Sr/Cu ratios of most samples between 1435 and 1270 m are larger than 5.0. Lower ratios occur at 1360 m and between 1321 and 1306 m. Thus, the palaeoclimate during the formation of the 1435–1270-m segment was hot and arid climate, interrupted by two cooling events represented by the rocks at 1306 m and between 1321 and 1306 m. The Sr/Cu ratios of the 1270–1100-m segment are mostly in a range between 1.3 and 5.0, and only larger than 5.0 at 1209, 1205, 1152, 1121 and 1106 m (Fig. 5). Hence, the climate conditions represented by the 1270–1100-m segment were mainly warm-humid, and characterized by only minor and relatively short-lived climate fluctuations. The FeO/MnO and Al_2_O_3_/MgO ratios show similar trends over the whole sequence which are opposite to the variations of the Sr/Cu ratios (Fig. 5). The negative correlation of the FeO/MnO and Al_2_O_3_/MgO ratios with the Sr/Cu ratios suggests that these ratios were controlled by the same geological process or processes. The Mg/Ca ratios show also a similar pattern as the FeO/MnO and Al_2_O_3_/MgO ratios, opposite to that of Sr/Cu ratios. Thus, the commonly seen positive correlation between Mg/Ca and Sr/Cu ratios is not applying here. However, we assume that the three corresponding FeO/MnO, Al_2_O_3_/MgO and Sr/Cu ratios are more robust indicators of geological significance than the single Mg/Ca ratio proxy. The late Cretaceous climate of the lower Zhoutian Formation in the Jitai Basin was mainly divided into a lower and upper part (Table 5 and Fig. 5). The lower part (1435–1270 m) experienced two cooling events and was characterized by wet-dry cycles, whereas the upper part (1270–1100 m) was dominated by warm-humid climate, overprinted by many minor climatic fluctuations.

### Palaeo-salinity

Boron is one of the most concentrated trace elements in sea water and it is mainly incorporated into the clay mineral illite in coastal to marine environments^26,27^. In contrast, Gallium is principally incorporated into terrestrial deposits, mainly in the form of smectite. According to studies of Wang et al.^28^ and Lan et al.^29^, the B/Ga ratio is a reliable indicator of salinity. Ratios lower than 3 indicate freshwater, ratios between 3 and 5 indicate brackish water, and ratios larger than 5 indicate salt water^30,31^.

The combination of SO_4_^2−^ in sea or saline water and Ba^2+^ in freshwater causes the precipitation of BaSO_4_. In contrast, Sr is assumed to migrate and precipitate in the center of the open marine basins or saline lakes as a result of the high solubility of SrSO_4_^32^. Therefore, Sr/Ba ratios can be used to distinguish marine and terrestrial environments. Overall, ratios exceeding 1 indicate sea or saline water, and ratios lower than 1 represent freshwater conditions^33–35^.

The B/Ga ratios in the lower Zhoutian Formation of the Jitai Basin range from 1.03 to 42.78 (Table 5). The B/Ga ratios show a similar pattern as the Sr/Cu ratios over the sequence which can be divided into two parts (Fig. 5). The B/Ga ratios are generally larger than 5 in the lower part from 1435–1270 m, indicating a saltwater environment. The ratios are mostly between 3–5 in the upper part above 1270 m, which includes also some samples with B/Ga ratios lower than 3 or higher than 5. The salinity during the formation of the upper part of the sedimentary sequence was mainly brackish, but including several changes to fresh/brackish and salt water environments. The Sr/Ba ratios range from 0.04 to 11.13 and their changes display similarities with those of the B/Ga and Sr/Cu ratios (Table 5). Compared with the B/Ga curve, the Sr/Ba curve follows more closely the Sr/Cu curve (Fig. 5). The similar patterns of the Sr/Cu and Sr/Ba ratios in the lower Zhoutian Formation implies that the salinity evolution in the basin was controlled by climate change. When the palaeoclimate as a whole was warm-humid, the salinity of the water was fresh/brackish, and when climate conditions were hot-arid, saline water prevailed in the basin. During the lower part (1435–1270 m) of the investigated lower Zhoutian Formation, salt water dominated in the Jitai Basin when hot-arid conditions prevailed, interrupted by fresh/brackish water periods during more warm-humid periods. During the formation of the upper part (1270–1100 m) of the investigated sequence, mainly fresh/brackish water existed in the basin under warm-humid climate conditions, interrupted by salt water periods when climate became hot and arid.

### Redox conditions

Several trace elements such as U, Ni, V, Mo, Cr and Co are commonly enriched in anoxic sediments because their solubility is controlled by the redox conditions^36–39^. In their study of late Jurassic redox environments in northwestern Europe, Jones & Manning^40^ concluded that the ratios of Ni/Co, V/Cr and V/(V + Ni) are reliable proxies of redox conditions. They established a set of trace-element indexes for the assessment of the redox environment (Table 6). Following their work, the trace-element ratios of Ni/Co, V/Cr and V/(V + Ni) were widely applied to reconstruct past redox conditions^3,40–43^.

**Table 6:** Geochemical proxies of redox environment.

Palec-oxygenation Facies	Oxygen Content (mL/L)	V/Cr	Ni/Co	V/(V + Ni)
Anaerobic, extremely dysaerobic	< 0.2	> 4.25	> 7.0	> 0.77
Dysaerobic, secondary aerobic	0.2–2.0	2.00–4.25	5.0–7.0	0.60–0.77
Aerobic	> 2.0	< 2.00	< 5.0	< 0.60

In addition, Cerium anomalies in REE distribution patterns are generally used to investigate the redox conditions^21,32,44–47^. Ce is present as Ce^3+^ under reducing conditions and separates from other REE^3+^ in the form of Ce^4+^ under oxidized conditions. The Ce/Ce* ratio is defined as 2(Ce)_PAAS_/((La)_PAAS_ + (Pr)_PAAS_), with _PAAS_ referring to the normalization of element concentrations against the PAAS. The ratios of Ce/Ce* can sensitively reflect the redox conditions in the sedimentary environment. A Ce/Ce* ratio larger than 1 or a positive anomaly indicates a reducing environment, whilst a ratio below 0.95 or a negative anomaly indicates an oxidized environment.

The Ni/Co ratios in the investigated lower Zhoutian Formation range from 1.23 to 6.97, the V/Cr ratios range from 0.24 to 2.21, the V/(V + Ni) ratios range from 0.43 to 0.88, and the Ce/Ce* ratios range from 0.70 to 1.07 (Table 5). All determined Ni/Co ratios are below 7.0, indicating an oxidation/weak oxidation environment. The Ni/Co ratios fluctuate over a wide range in the lower part (1435–1270 m), but even the maximum Ni/Co ratio of 6.97 indicates a weak oxidation environment. The Ni/Co ratios are relatively constant in the upper part (1270–1100 m), with values exceeding 5.0 only at 1121, 1106 and 1101 m. The V/Cr ratios are below 2.0 apart from a single sample collected from 1106 m core depth, indicating an oxidation environment too. Most of the V/(V + Ni) ratios are between 0.6 and 0.77, and only four exceed 0.77 of which three were sampled from the lower part at 1350, 1320 and 1315 m. Most of the Ce/Ce* ratios are around 0.95, but a significantly higher ratio of 1.07 was determined at 1284 m and a low ratio of 0.70 at 1115 m. However, all redox-condition proxies indicate that the rocks of the lower Zhoutian Formation formed in an oxidized environment, only affected by minor fluctuations in its lower part (1435–1270 m; Tables 5 and 6, Fig. 5).

### Provenance

The chemical composition of terrigenous clastic rocks is an integrated reflection of the nature, denudation and transport processes of the source area. The elements Cs, Zr, Th, Hf, Ti, La and Yb are relatively stable with respect to weathering, transportation and diagenesis. Because they are not intensively affected by migration, they are often used to assess the tectonic setting and the types of rocks in the source area^21,48–51^. The composition of REE may change slightly during deposition. The REE abundances in source rocks and the weathering conditions in source areas are the major factors controlling the REE in the accumulating sediments. Hence, REE in clastic sedimentary rocks are widely used as the main indicator to identify the provenance^21,52^. In general, the La-Th-Sc, the Th-Sc-Zr/10 and the La/Th-Hf compositions are used to identify the tectonic setting of the source area^49,53–55^. The La-Th-Sc discriminant diagram is predominantly used to distinguish between continental and oceanic island arcs, whilst the Th-Sc-Zr/10 discriminant diagram can be used to distinguish between active and passive continental margins.

**Figure 6:**
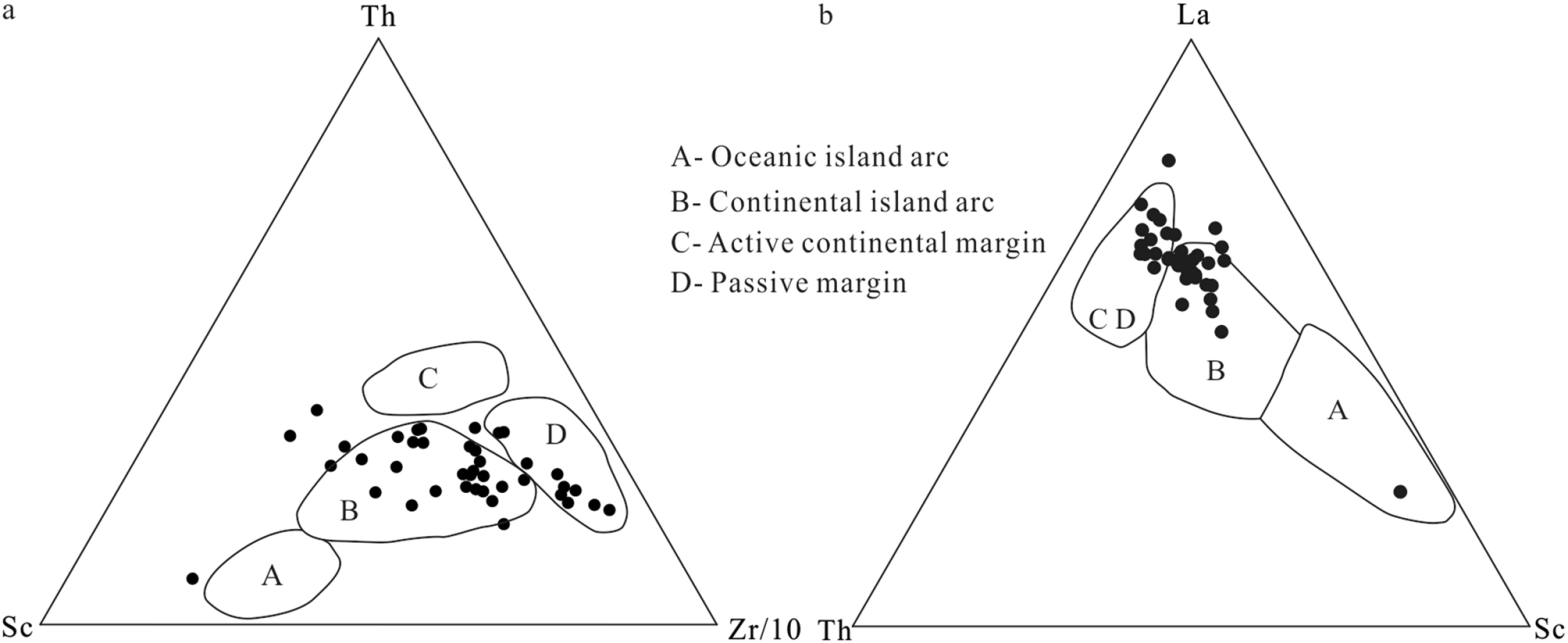
(**a**) Th-Sc-Zr/10 discriminatory plot (modified after Bhatia and Crook^49^) of late Cretaceous samples from the lower Zhoutian Formation, Jitai Basin. (**b**) La-Th-Sc discriminatory plot (modified after Bhatia and Crook^49^).

**Figure 7:**
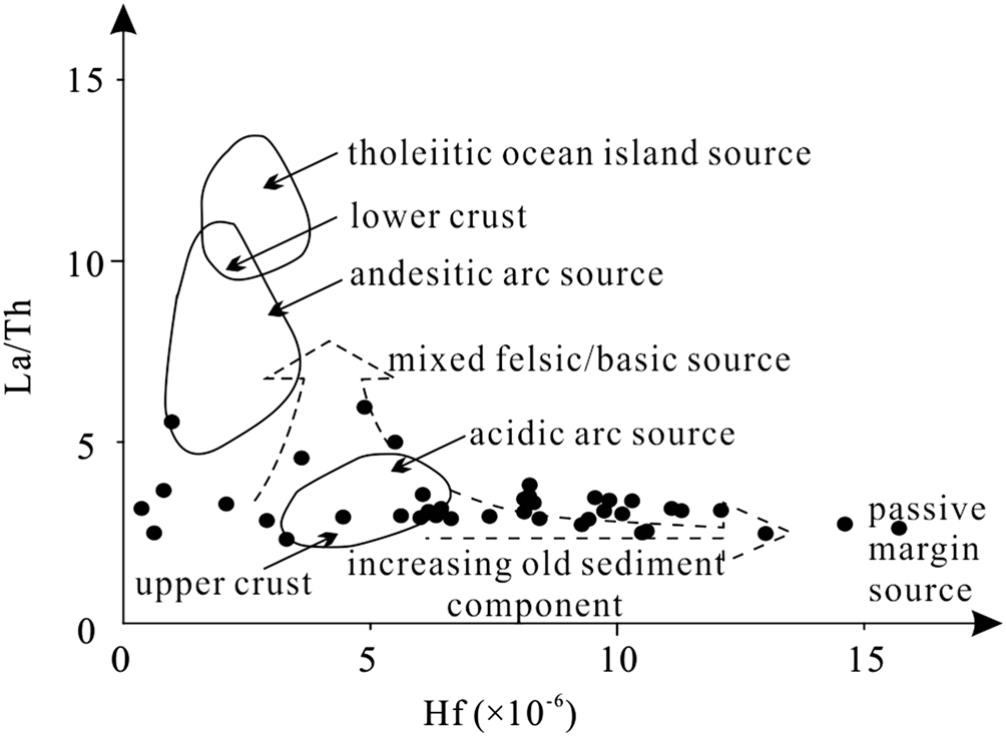
La/Th-Hf discriminatory plot (modified after Floyd et al.^54^) of late Cretaceous samples from the lower Zhoutian Formation, Jitai Basin.

The samples from the lower Zhoutian Formation mainly cluster in the continental island arc field in the Th-Sc-Zr/10 discriminant diagram, a minority falls into the field of the passive margin, and a few samples are located outside the four predefined fields (Fig. 6a). In the La-Th-Sc discriminant diagram, most of the samples are located in the continental island arc field, some in the region of active/passive continental margins, and only one sample in the oceanic island arc field (Fig. 6b). In La/Th-Hf discriminant diagram, most of the samples are located in the field of the increasing old sediment component, some in the regions of the felsic and the mixed felsic/basic sources, and only one sample in the field of the andesitic arc source (Fig. 7). The Th/Sc ratios range from 0.58 to 3.23, with an average of 1.31 (Table 5). Most of the Th/Sc ratios are located in the range of felsic rocks (0.84–20.5) and are significantly larger than those of mafic rocks (0.05–0.22). The La/Sc ratios range from 0.33 to 11.51, with an average of 4.20 (Table 5). Similar to the Th/Sc ratios, the La/Sc ratios mostly lie within the felsic rock range (2.5–16.3)^56–58^. Hence, most of the samples from the lower Zhoutian Formation were formed in a continental island arc or passive margin environment. The sediments’ provenance is mostly mixing of felsic material of the upper continental crust and old sediments.

## Conclusions

This study represents a first systematic investigation of major, trace and rare elements of the late Cretaceous lower Zhoutian Formation in the Jitai Basin. The main conclusions are the following:

The climate history during the formation of the sedimentary sequence is divided into a lower and an upper part. The lower part (1435–1270 m) was dominated by hot-arid climate, interrupted by two cooling events. In contrast, warm-humid climate prevailed during the formation of the upper part (1270–1100 m), further characterized by frequent climate fluctuations.

The salinity in the Jitai Basin was high during the formation of the lower part, and it was reduced to fresh/brackish conditions during two cooling events. During the formation of the upper part, fresh/brackish water conditions prevailed, interrupted by saline water periods during hot-arid climate conditions. Sediment accumulation during the late Cretaceous lower Zhoutian Formation occurred in an oxidized environment, with periods of weaker oxidation during the formation of its lower part.

The sediments of the lower Zhoutian Formation in the Jitai Basin were mainly deposited in a continental island arc to passive margin setting. Their provenance was a mixture of upper crust felsic sediments and old sediment components.

## References

[1] Nameroff TJ, Calvert SE, Murray JW (2004). Glacial–interglacial variability in the eastern tropical North Pacific oxygen minimum zone recorded by redox-sensitive trace metals. Paleoceanography.

[2] Tribovillard N, Averbuch O, Deleeschouwer X, Racki G, Riboulleau A (2004). Deep–water anoxia over the Frasnian-Famennian boundary (La Serre, France): A tectonically–induced oceanic anoxic event?. Terra Nova.

[3] Tribovillard N, Algeo TJ, Lyons T, Riboulleau A (2006). Trace metals as paleoredox and paleoproductivity proxies: An update. Chem. Geol..

[4] Zhang L (2016). Geochemistry of sediments from the Huaibei Plain (east China): Implications for provenance, weathering, and invasion of the Yellow River into the Huaihe River. J. Asian Earth Sci..

[5] Wei GJ, Liu Y, Li XH, Shao L, Fang DY (2004). Major and trace element variations of the sediments at ODP Site 1144, South China Sea, during the last 230 ka and their palaeoclimate implications. Palaeogeogr. Palaeoclimatol. Palaeoecol..

[6] Ren JS (1991). On the geotectonics of southern China. Acta Geol. Sin. Engl..

[7] Zhao Y (2004). Yanshanian movement and conversion of tectonic regimes in East Asia. Earth Sci. Front..

[8] Dong SW (2008). Jurassic tectonic revolution in China and new interpretation of the “Yanshan movement”. Acta Geol. Sin..

[9] Dong SW (2010). The tectonic stress field in the Dabashan orogen resulting from late Mesozoic intra-continental orogeny. Acta Geol. Sin..

[10] Rodríguez-López JP, Wu CH (2010). Recurrent deformations of aeolian desert dunes in the Cretaceous of the South China Block: Trigger mechanisms variability and implications for aeolian reservoirs. Mar. Pet. Geol..

[11] Fang ZX (2006). Sedimentary Filling Model of Salt Lake in the Janghan Basin.

[12] Yao QC, Lou JS (2008). An analysis of hydrocarbon pooling conditions in Yuanjiang sag, the Dongting basin. Nat. Gas Ind. (Chengdu, China).

[13] Liu CL, Wang ML, Jiao PC, Chen YZ (2009). The probing of regularity and controlling factors of potash deposits distribution in Lop Nur salt lake, Xinjiang. Acta Geosci. Sin..

[14] Liu CL (2013). Characteristics and formation of potash deposits in continental rift basins: A review. Acta Geosci. Sin..

[15] Liu CL (2006). Studies of fluid inclusions in glauberite of middle upper Pleistocene strata and their paleoclimatic significance in lop Nur salty lake, Xinjiang, NW China. Acta Mineral. Sin..

[16] Liu CL, Jiao PC, Wang ML (2010). A tentative discussion on exploration model for potash deposits in basins of China. Miner. Deposits (Beijing, China).

[17] Sun XH (2017). Paleoclimatic information recorded in fluid inclusions in halites from Lop Nur, Western China. Sci. Rep..

[18] Yu XQ, Shu LS, Deng GH, Wang B, Zu FP (2005). Geochemical Features and tectonic significance of the alkaline-basalts from Ji’an-Taihe Basin, Jiangxi province. Geoscience (Beijing, China).

[19] Lu QY (1991). Sedimentary characteristics of Cretaceous in the Taihe depression, Jitai Basin, Jiangxi. Geophys. Geochem. Explor..

[20] Zhou MJ, Hu L, Huang XN, Han XP (2017). Metallogenic geological characteristics and prospect of development and utilization of Meigang Li-bearing Brine deposit in the Taihe County, Jiangxi Province. Mod. Min..

[21] Taylor SR, McLennan SM (1985). The Continental Crust: Its Composition and Evolution, an Examination of the Geochemical Record Preserved in Sedimentary Rocks.

[22] Evensen NM, Hamilton PJ, Onions RK (1978). Rare-earth abundances in chondritic meteorites. Geochim. Cosmochim. Acta.

[23] Lerman A (1978). Lakes: Chemistry, Geology, Physics.

[24] Liu G, Zhou DS (2007). Application of micro elements analysis in identifying sedimentary environment. Exp. Pet. Geol..

[25] Song MS (2005). Sedimentary environment geochemistry in the Shasi section of Southern Ramp, Donying depression. J. Mineral. Petrol..

[26] Couch EL (1971). Calculation of paleosalinities from boron and clay mineral data. Am. Assoc. Pet. Geol. Bull..

[27] Dominik J, Stanley DJ (1993). Boron, beryllium and sulfur in Holocene sediments and peats of the Nile delta, Egypt: Their use as indicators of salinity and climate. Chem. Geol..

[28] Wang YY, Guo WY, Zhang GD (1979). Application of some pollen spore assemblage and its significance of stratigraphy and paleogeographic change in the Yangtze delta. Oceanologia.

[29] Lan HX, Ma DX, Xu MG, Zhou QW, Zhang GW (1987). Some geochemical indicators of the Pearl River Delta and their facies significance. Mar. Geol. Q. Geol..

[30] Hu XF (2012). Trace element characteristics of Eocene Jijuntun Formation and the favorable metallogenic conditions of oil shale in Fushun Basin. J. Jilin Univ. Earth Sci. Ed..

[31] Zhang MM, Liu ZJ, Xu SC, Sun PC, Hu XF (2013). Element response to the ancient lake information and its evolution history of argillaceous source rocks in the Lucaogou Formation in Sangonghe Area of Southern Margin of Junggar Basin. J. Earth Sci..

[32] Cao J (2012). Trace and rare earth element geochemistry of Jurassic mudstones in the northern Qaidam Basin, northwest China. Chem. Erde.

[33] Deng HW, Qian K (1993). Sedimentary Geochemistry and Environmental Analysis.

[34] Wang AH (1996). Discriminant effect of sedimentary environment by the Sr/Ba ratio of different existing forms. Acta Sedimentol. Sin..

[35] Peng SZ (2016). Geochemical and grain–size evidence for the provenance of loess deposits in the Central Shandong Mountains region, northern China. Quatern. Res..

[36] Francois R (1988). The study on the regulation of the concentrations of some trace metals (Rb, Sr, Zn, Cu, V, Cr, Ni, Mn and Mo) in Saanich inlet sediments, British Columbia, Canada. Mar. Geol..

[37] Arthur MA, Sageman BB (1994). Marine black shales: Depositional mechanisms and environments of ancient deposits. Annu. Rev. Earth Planet. Sci..

[38] Yuri ZN, Eder VG, Zamirailova AG (2008). Composition and formation environments of the Upper Jurassic-Lower Cretaceous black shale Bazhenov Formation (the central part of the West Siberian Basin). Mar. Pet. Geol..

[39] Hetzel A, März C, Vogt C, Brumsack HJ (2011). Geochemical environment of Cenomanian-Turonian black shale deposition at Wunstorf (northern Germany). Cretaceous Res..

[40] Jones B, Manning DAC (1994). Comparison of geochemical indices used for the interpretation of palaeoredox conditions in ancient mudstones. Chem. Geol..

[41] Dill H (1986). Metallogenesis of early Paleozoic graptolite shales from the Graefenthal Horst (Northern Bavaria-Federal Republic of Germany). Econ. Geol..

[42] Calvert SE, Pedersen TF (1993). Geochemistry of recent oxic and anoxic marine sediments: Implications for the geological record. Mar. Geol..

[43] Algeo TJ, Maynard JB (2004). Trace-element behavior and redox facies in core shales of upper Pennsylvanian Kansas-type cyclothems. Chem. Geol..

[44] Zhao ZH (1997). Principles of Trace Element Geochemistry.

[45] Holser WT (1997). Evaluation of the application of rare-element elements to paleoceanography. Palaeogeogr. Palaeoclimatol. Palaeoecol..

[46] Shields G, Stille P (2001). Diagenetic constraints on the use of cerium anomalies as palaeoseawater redox proxies: An isotopic and REE study of Cambrian phosphorites. Chem. Geol..

[47] Teng G, Liu WH, Xu YC, Chen JF (2005). Correlative study on parameters of inorganic geochemistry and hydrocarbon source rocks formative environment. Adv. Earth Sci..

[48] McLennan SM (1989). Rare earth elements in sedimentary rocks: Influence of provenance and sedimentary processes. Rev. Mineral. Geochem..

[49] Bhatia MR, Crook KA (1986). Trace element characteristics of graywackes and tectonic setting discrimination of sedimentary basins. Contrib. Mineral. Petrol..

[50] Girty GH, Hanson AD, Knaack C, Johnson D (1994). Provenance determined by REE, Th, and Sc analyses of metasedimentary rocks, Boyden Cave roof pendant, central Sierra Nevada, California. J. Sediment. Res..

[51] Garver JI, Scott TJ (1995). Trace elements in shale as indicators of crustal provenance and terrane accretion in the southern Canadian Cordillera. Geol. Soc. Am. Bull..

[52] Bhatia MR, Taylor SR (1981). Trace-element geochemistry and sedimentary provinces: A study from the Tasman Geosyncline, Australia. Chem. Geol..

[53] Bhatia MR (1983). Plate tectonics and geochemical composition of sandstones. J. Geol..

[54] Floyd PA, Leveridge BE (1987). Tectonic environment of the Devonian Gramscatho basin, south Cornwall: Framework mode and geochemical evidence from turbidite sandstones. J. Geol. Soc. (London, U.K.).

[55] Shao L, Stattegger K, Garbe-Schoenberg C (2001). Sandstone Petrology and Geochemistry of the Turpan Basin (NW China): Implications for the Tectonic Evolution of a Continental Basin. J. Sediment. Res..

[56] Cullers RL (1994). The controls on the major and trace element variation of shales, siltstones, and sandstones of Pennsylvanian-Permian age from uplifted continental blocks in Colorado to platform in Kansas, USA. Geochim. Cosmochim. Acta.

[57] Cullers RL (2000). The geochemistry of shales, siltstones and sandstones of Pennsylvanian-Permian age, Colorado, USA: Implications for provenance and metamorphic studies. Lithos.

[58] Cullers RL, Podkovyrov VN (2000). Geochemistry of the Mesoproterozoic Lakhanda shales in southeastern Yakutia, Russia: Implications for mineralogical and provenance control, and recycling. Precambrian Res..

